# Associations between metabolic syndrome and clinical benign prostatic hyperplasia in a northern urban Han Chinese population: A prospective cohort study

**DOI:** 10.1038/srep33933

**Published:** 2016-09-22

**Authors:** Si-Cong Zhao, Ming Xia, Jian-Chun Tang, Yong Yan

**Affiliations:** 1 Department of Urology, Beijing Shijitan Hospital, Capital Medical University, Beijing, China; 2 Department of Cardiology, Beijing Shijitan Hospital, Capital Medical University, Beijing, China

## Abstract

Biologic rationales exist for the associations between metabolic syndrome (MetS) and benign prostatic hyperplasia (BPH). However, epidemiologic studies have yield inconsistent results. The aim of the present study was to prospectively evaluate the associations of MetS with the risk of BPH. The presence of MetS, the number of MetS components, and the individual MetS components were evaluated. After adjusting for potential confounders, MetS was associated with increased risk of BPH (HR: 1.29; 95% CI, 1.08–1.50; *p* < 0.001). Compared with subjects without any MetS components, the HRs were 0.88 (95% CI, 0.67–1.09; *p* = 0.86), 1.18 (95% CI, 0.89–1.47; *p* = 0.29) and 1.37 (95% CI, 1.08–1.66; *p* = 0.014) for subjects with 1, 2, or ≥3 MetS components, and there was a biologic gradient between the number of MetS components and the risk of BPH (*p*-trend < 0.001). Central obesity and low high-density lipoprotein cholesterol were the two main divers of the associations between these two conditions, with HRs of 1.93 (95% CI, 1.14–2.72; *p* = 0.001) for central obesity, and 1.56 (95% CI, 1.08–2.04; *p* = 0.012) for low HDL-C. Our findings support the notion that MetS may be an important target for BPH prevention and intervention.

Benign prostatic hyperplasia (BPH), a condition characterized by the development of hyperplasic nodules mainly in the transition zone of the prostate, the overall prostatic enlargement, and the related lower urinary tract symptoms (LUTS), is highly prevalent among middle-aged and elderly men^1^. Historically, aging and androgens were considered the two principal determinants of the risk of BPH; however, etiologies and natural history of BPH are not well defined. Because emerging evidence indicates that a considerable number of modifiable risk factors such as obesity and lack of physical activity have an important role in the BPH etiology^2^,^3^,^4^, it is increasingly recognized that BPH could partly be a preventable disease^5^. These findings also highlighted that a cost-effective way for addressing BPH could be to intervene with these modifiable risk factors before disease development or treatment becomes necessary. Thus, both clinicians and scientists have begun to search for multiple potential modifiable risk factors, in the hopes of identifying adjuvant care approaches to prevent BPH.

As a part of this strategy, preclinical and clinical evidence considering the connections between metabolic syndrome (MetS) and BPH have also attracted attentions in recent years. In brief, MetS describes a complex of disorders related to metabolic aberrations, comprising central obesity, hypertension, dyslipidemia, insulin-resistance with compensatory hyperinsulinemia, and glucose intolerance that increase an individual’s risk of cardiovascular disease and type 2 diabetes, with the essence of MetS lies in the combination or clustering of these metabolic risk factors^6^. At the molecular and biochemical level, several systemic perturbations associated with MetS, including the systemic pro-inflammatory state, sex steroids alterations, elevated insulin-like growth factor, and autonomic hyperactivity, have been implicated in the course of BPH^7^. Conversely, results from epidemiological studies; however, although generally in favor of a association between MetS and BPH, were somewhat conflicting^8^,^9^. Furthermore, if MetS influences BPH through its effects on sex steroids, as has been suggested^10^, the associations between MetS and BPH may differ with races. In this context, former studies that performed on Asian populations have largely revealed a null or even inverse association between MetS and BPH and/or its related LUTS^11^,^12^,^13^,^14^,^15^.

On the other hand, although there was a strong argument for biologic plausibility between these MetS and BPH, most epidemiological studies in this field, either revealed a positive, null or even inverse association were cross-sectional in design and were therefore lack of adequate strength to determine whether MetS induces BPH or whether these two entities simply occur as a common phenomenon of aging. Thus far, a well-designed prospective cohort for determining causality between MetS and BPH is lacking in the first place. However, given the epidemic of MetS, if MetS does increase the risk of BPH, realization and utilization of their associations would have profound implications in decreasing the public health burden of BPH.

In the present study, our overall objective was to prospectively evaluate the associations between MetS and the risk of BPH in men participated in routine health check-ups. More importantly, our specific objectives were to determine if the individual components of MetS are associated with the risk of BPH, and to determine if there is a biologic gradient in the association between the number of MetS components and the risk of BPH.

## Methods and Materials

### Study population

Based on the information of routine health check-ups by participants at the physical examination center of Beijing Shijitan Hospital, we consecutively set up a prospective cohort and conducted follow-up between April 2006 and December 2015 in a northern urban Han Chinese population. A total of 1,869 men aged 45–73 years who participated in the routine health check-ups at least two times since April 2006 were included in this study. Among these participants, 739 men were excluded based on the following exclusion criteria: 98 had a prior history of urogenital tumor; 56 had a prior history of urethral stricture; 121 had neurogenic or overactive bladder (OAB); 422 men reported a documented prostate enlargement, International Prostate Symptom Score (I-PSS) >7, or had a prior history of treatment for BPH (α-blockers, anticholinergics, 5α-reductase inhibitors, phosphodiesterase-5 inhibitors or surgical). In addition, 102 participants regularly took diuretics for hypertension, congestive heart failure, or renal function insufficiency, were also excluded from the present study. Because certain participants met more than one of this exclusion criteria, the final subjects analyzed herein was 1,130. The total follow-up time was 5,312.8 person-years, and the median follow-up time was 7.7 person-years (interquartile range [IQR]: 4.3–9.7). This study was approved by the institutional review boards of Capital Medical University, and all the participants entering the cohort provided written informed consent. This study did not required any deviation of the current clinical practice and was conducted in accordance with the principles of research involving human subjects as expressed in the Declaration of Helsinki (64th, 2013) and with Good Clinical Practice standard (GCP).

### Data collection and measurements

The routine health check-ups were performed after an overnight fasting of at least 12 hours. All the participants underwent a baseline survey that focused on anthropometric, laboratory, lifestyle, and socio-demographic characteristics, as well as the baseline BPH components measurements. Height (cm), weight (kg), waist circumference (cm), and blood pressure (mmHg) which included systolic blood pressure and diastolic blood pressure were measured by nurses following a standard protocol from the WHO report^16^. Body mass index (BMI) was calculated as weight divided by the square of the height (kg/m^2^). In addition, prostate volume (PV) was determined by suprapubic ultrasonography (3.5 MHz, Hitachi EUB-400) using the formula for an elliptical volume (height × width × length × π/6). Suprapubic ultrasonographies with PV measurements were performed by a single high-volume radiologist, who performs approximately 1,800 such examinations every year. Maximum urinary flow rate (Q_max_) was measured by uroflowmetry at a voided volume >150 mL.

Before undergoing digital rectal examinations, a 10mL of fasting venous blood sample was drawn by venipuncture when the subject had been sedentary in a sitting or supine position for at least 15 minutes. Blood biochemical analyses, included fasting plasma glucose (FPG), triglycerides, high-density lipoprotein cholesterol (HDL-C), low-density lipoprotein cholesterol (LDL-C), and total cholesterol, were performed using an automatic Biochemical Analyzer (Hitachi 7020, Tokyo, Japan). Serum prostate-specific antigen (PSA) levels were determined by using the monoclonal Tandem-R kit (Hybritech Inc; San Diego, CA, USA).

At the time of enrollment, information about marital status, socio-economic status, smoking status, alcohol consumption, physical activity, sexual activity, comorbidities, and medications usage were collected through a structured standardized questionnaire with a field research assistant present. In detail, marital status was categorized as married and living with their spouse vs. not married or widowed. Socio-economic status (SES) index was measured as a combination of education and household income and which categorized as low (lower 25% of the distribution), middle (middle 50% of the distribution) and high (upper 25% of the distribution)^17^. While the physical activity was determined using the 12-item Physical Activity Scale for the Elderly (PASE)^18^, which was further categorized as low (≤100), medium (100–249) and high (≥250). Smoking status and alcohol consumption were both categorized as never, ex-user and current use. Additionally, sexual activity was considered active if the subject experienced at least one episode of intercourse during the last month, and the opposite condition was considered inactive. All the subjects also completed a Chinese version of International Prostate Symptom Sore (I-PSS) questionnaire of which the questions were similar to those in the American Urological Association Symptom Index^19^,^20^, with an additional question for assessing the quality of life due to urinary symptoms.

### Definition of metabolic syndrome

The presence and severity of MetS were evaluated at the time of enrollment. Our definition of MetS was in accordance with the most recent joint consensus statement from the American Heart Association/National Heart, Lung, and Blood Institute (AHA/NHLB) and International Diabetes Federation (IDF)^6^. According to this criteria, MetS was defined as the simultaneous occurrence of at least three of the following five metabolic aberrations: (1) waist circumference ≥85 cm for Chinese males^21^; (2) triglycerides ≥150 mg/dL or drug treatment for elevated triglycerides; (3) HDL-C <40 mg/dL or drug treatment for reduced HDL-C; (4) elevated blood pressure (systolic BP ≥130 mm Hg and/or diastolic BP ≥85 mm Hg) or antihypertensive drug treatment in a patient with a history of hypertension; and (5) FPG ≥100 mg/dL or drug treatment for elevated glucose. MetS was analyzed dichotomously and also as a four-level categorical variable (based on the number of MetS components present: 0, 1, 2, or ≥3).

### Definition of clinical BPH

As there was no widely accepted definition of clinical BPH available for epidemiological studies, we defined subjects with clinical BPH at follow-up *as per* the 2011 Chinese Guideline for BPH^22^, by using the data of routine health check-ups. According to this guideline, criteria for defining clinical BPH included: (1) male aged ≥45 years; (2) sustained LUTS (I-PSS >7); (3) prostate enlargement (TPV ≥31 mL); and (4) repeated Qmax <15 ml/s. Furthermore, based on the medical records reviewing during the follow-up period, we introduced a potentially more stringent definition, “the clinically significant BPH”, which was defined as taking medication (α-blockers, anticholinergics and/or 5α-reductase inhibitors) or histories of surgery (TURP) for BPH. Thus, the clinical BPH was defined as to *either* a clinical diagnosis of BPH *or* histories of specific treatment for BPH. Although histological data confirming the presence of BPH was not available in our cohort, this combined criteria for defining clinical BPH was closely similar to that used in recent epidemiological studies^23^,^24^,^25^. Therefore, our definitions are practical and useful for translation of results into clinical practice and for comparison of results across other studies in this field.

### Endpoints measurements

In the present study, failure events in survival analyses included: (1) a clinical BPH identification (*either* a clinical diagnosis of BPH *or* histories of specific treatment for BPH; 429 cases); (2) a histological confirmation of prostate cancer (PCa; 32 cases); and (3) deceased for any causes (27 cases). The follow-up period was calculated as the interval from April 2006 to the clinical BPH identification, PCa diagnosis, death, lost to follow-up, or December 2015, whichever occurred earlier. Of note, besides dates on clinical diagnosis of BPH were recorded in accordance with the date of the corresponding health check-up (assumed this happened at the end of this follow-up), dates on the other failure events were directly abstracted from medical records. In addition, in case of censoring it was assumed that this event happened at the end of last known follow-up (lost to follow-up, 22 cases). The information on outcomes was updated annually.

Specifically, PCa was diagnosed in 32 subjects at follow-up. In detail, a total of 158 subjects met with a serum PSA >4 ng/mL and/or palpable prostatic nodules. 132 subjects visited the Urologic Outpatient of Beijing Shijitan Hospital and 86 subjects among them who were considered at high risk of harboring PCa underwent transrectal ultrasound (TRUS)-guided prostate biopsy. Finally, 32 subjects were diagnosed with PCa by biopsies.

### Statistical analysis

Baseline cohort characteristics were compared between subjects with and without MetS, by using the Mann-Whitney U test, student-t test, or chi-square test as appropriate. Considering age was an established risk factor for BPH, the Mantel-Haenzsel extension test for trend was used to test the linear trend across different age categories on the risk of clinical BPH. The total person-years of follow-up were calculated as the sum of the follow-up period from the baseline to the clinical BPH identification or to the endpoints previously listed.

The associations between the presence of MetS and the number of MetS components and clinical BPH development were first evaluated with the chi-square test and Mantel-Haenzsel extension test for trend, respectively. Afterwards, the independent associations between the presence of MetS, the number of MetS components, and the risk of clinical BPH were evaluated with a multivariable Cox proportional hazards model with adjusting for age, marital status, socio-economic status, smoking status, alcohol consumption, physical activity, sexual activity, BMI, total cholesterol, LDL-C, comorbidities including diabetes, congestive heart disease (CVD), subclinical ischemic stroke and autoimmune disease, and the baseline BPH components measurements (i.e. PV, Q_max_, I-PSS and serum PSA levels). Sensitivity analyses were performed by examining the full range of number of MetS components (i.e. 1, 2, 3, 4 and 5) with the risk of clinical BPH. The Cox proportional hazard ratios (HRs) and 95% confidence intervals (95% CIs) were calculated.

To assess the independent effect of each individual component of MetS on the risk of clinical BPH, a similar multivariable Cox proportional hazards model were further constructed by including all the five components of MetS in a mutually adjusted state for each and along with the confounding factors previously listed. To analyze the change in risk of clinical BPH in terms of the extent of central obesity, study subjects were further categorized into three subgroups according to the 90- and 102 cm cut off points of waist circumference (WC), which are commonly used in Asian and Western populations^26^. To more specifically examine the association between low HDL-C and the risk of clinical BPH, study subjects were also divided into three subjects according to measure HDL-C, i.e. >60 mg/dL, 40–60 mg/dL and <40 mg/dL, consistent with the definition established by the Chinese Medical Association to measure the difference in risk of clinical BPH in these subgroups^27^. For all the models, specificity analyses were performed by redoing the statistics aimed at the clinically significant BPH solely. Statistical model assumptions were also verified for all the models. Data were analyzed using the SPSS software version 13.0 for windows (SPSS Inc., Chicago, Ill., USA). Two-tailed *p*-values of < 0.05 were considered to be statistically significant.

## Results

### The baseline characteristics

The total follow-up period was 5,312.8 person-years. Of 1,130 subjects, 258 (22.8%) subjects had MetS at baseline, and 429 (38.0%) subjects developed clinical BPH at follow-up. The incidence of clinical BPH was higher in men with MetS (55.0%, 142/258) than in men without MetS (32.9%, 287/872). The baseline characteristics of the subjects grouped by MetS status were demonstrated in Table 1. In addition to being older at baseline, subjects with MetS were more likely to be general obese, to be married and living with their spouse, to be sexually inactive, and in higher rank of SES status. As we expected, subjects with MetS were much more likely to be affected by diabetes and CVD, although the prevalence of other comorbidities like subclinical ischemic stroke and autoimmune diseases were not different between the two groups. However, with regards to the BPH components measurements, i.e. PV, Q_max_, serum PSA levels and I-PSS-QoL, there were no significant differences between subjects with and without MetS at baseline (all *p* > 0.05).

### The incidence of clinical benign prostatic hyperplasia at follow-up

The median age at the identification of clinical BPH was 54.0 years (IQR: 51.0–58.0). In the 429 subjects who had developed clinical BPH during the follow-up, 259 cases were initially identified by clinician diagnosis at the routine health check-ups; based on medical records reviewing, the remaining 170 cases were identified by having histories of specific treatment for BPH. As shown in Table 2, the incidence of the overall clinical BPH increased significantly with age at baseline, from 24.1% among subjects aged 45–49 years to 74.8% among subjects aged ≥60 years, while even more increased manner was seen in the incidence of the clinically significant BPH with older age categories (both *p*-trend < 0.001). As for MetS and its individual components, their baseline prevalence also significantly increased with age (all *p*-trend < 0.001).

### Associations of metabolic syndrome with the risk of clinical benign prostatic hyperplasia

**Figure 1:**
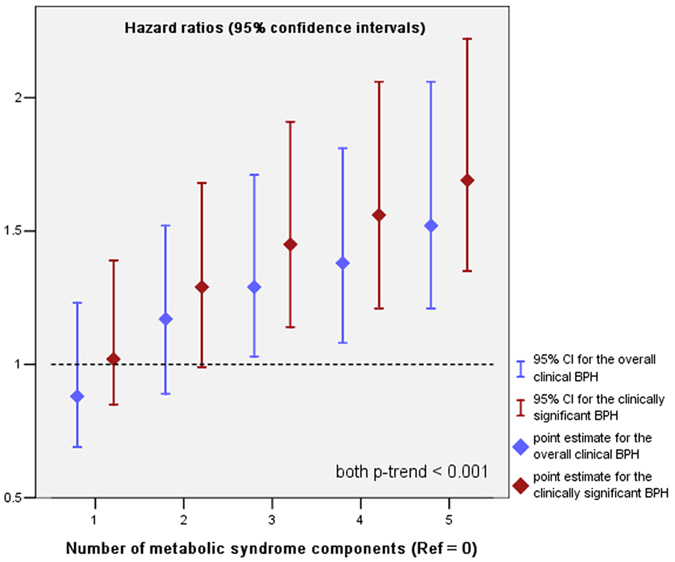
The risk of clinical benign prostatic hyperplasia according to the number of metabolic syndrome components. Hazard ratios (HRs) and 95% confidence intervals (95% CIs) were calculated by multivariable Cox proportional hazards analyses with adjusting for the following confounding factors: age, marital status, socio-economic status, smoking history, alcohol usage, physical activity, sexual activity, BMI, total cholesterol, LDL-C, comorbidities which included diabetes, CVD, subclinical ischemic stroke and autoimmune disease, and the baseline BPH components measurements which included PV, Q_max_, IPSS and serum PSA levels. Abbreviations: Ref = the reference category.

In the multivariable Cox proportional hazards models, although having one component of MetS was neither associated with increased risk of the overall clinical BPH nor the clinically significant BPH, an increasing number of MetS components was significantly and linearly associated with a higher probability of developing clinical BPH, irrespective of the outcome definitions based on (Table 3). [Table t4]Having ≥3 vs. 0 components of MetS was associated with a 37% increased risk of the overall clinical BPH (HR: 1.37; 95% CI, 1.08–1.66; *p* = 0.014), and was even associated with a 58% increased risk of the clinically significant BPH (HR: 1.58; 95% CI, 1.26–1.90; *p* = 0.001). Interestingly, although having 2 vs. 0 components of MetS did not showed any differential effect on the risk of the overall clinical BPH, subjects with 2 components of MetS was associated with a 31% increased risk of the clinically significant BPH (HR: 1.31; 95% CI, 1.08–1.54; *p* = 0.039). Moreover, when examining the full range of number of MetS components, there was a significant biologic gradient between the number of positive MetS components and the risk of clinical BPH, irrespective of the outcome definitions based on (Fig. 1; both *p*-trend < 0.001). Although the dichotomous comparisons for the presence of MetS were also significant and still irrespective of the outcome definitions based on (both *p* < 0.001), the magnitudes of associations were lowered in such a dichotomous manner, which was likely due to the subjects without MetS unavoidably included a number of patients had 1 or 2 MetS components while not meet the criteria for MetS.

### Associations of the individual components with the risk of clinical benign prostatic hyperplasia

**Figure 2:**
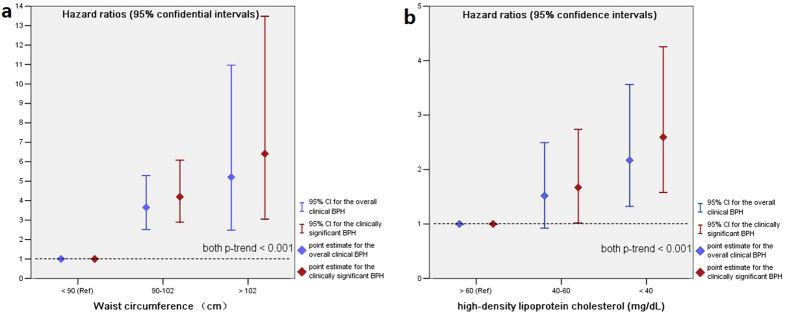
The risk of clinical benign prostatic hyperplasia according to the level of WC (**a**) and HDL-C (**b**). Hazard ratios (HRs) and 95% confidence intervals (95% CIs) were calculated by multivariable Cox proportional hazards analyses with adjusting for all the dichotomous components of metabolic syndrome in a mutually adjusted state for each and along with the other confounding factors: age, marital status, socio-economic status, smoking history, alcohol usage, physical activity, sexual activity, BMI, total cholesterol, LDL-C, comorbidities which included diabetes, CVD, subclinical ischemic stroke and autoimmune disease, and the baseline BPH components measurements which included PV, Q_max_, IPSS and serum PSA levels. Abbreviations: Ref = the reference category.

Among the individual MetS components, central obesity and low HDL-C were independently associated with increased risk of clinical BPH, after adjusting for the other MetS components in a mutually adjusted state for each and along with the other confounding factors previously listed, with multivariable HRs of 1.93 (95% CI, 1.14–2.72; *p* = 0.001) for the central obesity, and 1.56 (95% CI, 1.08–2.04; *p* = 0.012) for the low HDL-C (Table 4). Finally, we examined the specific associations between the risk of clinical BPH and obesity and low HDL-C. In these analyses, subjects were classified into three subgroups by WC and HDL-C, rather than undergoing analysis as a single dichotomous group. Figure 2 shows the risk of clinical BPH by WC and HDL-C subgroups. Even after fully adjusting for a broad range of potential confounding factors, the risk of clinical BPH significantly and linearly increased as WC increased; on the other hand, a significant inverse linear association was observed between HDL-C and the risk of clinical BPH (Fig. 2a,b; both *p*-trend < 0.001), and the HRs of central obesity was much more greater than that for low HDL-C. Once again, these specific associations were still irrespective of the outcome definitions and were even more stronger aimed at the clinically significant BPH.

## Discussion

Over 10 years of follow-up, data from this cohort study of Chinese aging men indicated that men with MetS or its certain components were more likely to develop clinical BPH after controlling for a wide range of potential confounding factors. More importantly, our study adds to demonstrate a significant biologic gradient between the full range of number of MetS components and the risk of clinical BPH. Among the individual MetS components, central obesity and low HDL-C were the main divers of the associations between these two entities. Of note, all the associations were even more stronger aimed at the more stringent outcome definition, i.e. clinically significant BPH. Although definitive proof of a causal association is hindered by the inability to randomize men to MetS vs. no MetS; however, given the prospective design and setting of our study that avoided the temporal ambiguity inherent in cross-sectional studies, our findings strengthen the argument for a causal relationship between MetS and BPH according to the Bradford-Hill criteria^28^.

As described earlier, although there were several studies reporting the association between MetS and BPH and/or its related LUTS, most of these studies used a cross-sectional design based on data collected at a certain time point and have yielded inconsistent results. Generally, there were two types of studies in this field, that were studies performed on healthy men merely focused on I-PSS based LUTS, and studies performed on patients with confirmed BPH/BPE. Interestingly, heterogeneous results were mostly reported in the association between MetS and LUTS in the general population. In this context, most studies that performed on American or European populations have demonstrated a direct association; however, studies with similar design that performed on Asian populations have reported null or even inverse associations^11^,^12^,^13^,^14^,^15^. Reasons for the heterogeneous results are unclear, but may involve different risk profiles, cultural backgrounds and specific health-care delivery system across populations^29^. Additionally, MetS was commonly analyzed in a binary manner in the former literature; however, we found that difference between having ≥3 vs. 0 components of MetS were greater than the dichotomous comparisons for the presence of MetS, thus it could be speculated that the inclusion of subjects with 1 or 2 MetS components in the control group can bias results toward the null^30^. On the other hand, most studies conducted in Asia unavoidably used weak data for study populations with a limited number of patients with clinically significant LUTS, thus they may not be adequately powered to identify a direct association. Further consideration is that it is now recognized that the etiology of LUTS is rather complex and not necessarily related to pathologies of the prostate. In fact many men with BPH do not suffer from LUTS and many men with LUTS do not present with BPH^31^, thus the associations with LUTS did not actually reflect the true associations with BPH.

By the contrast, literature addressing the associations between MetS and the BPH components measurements in patients with confirmed BPH/BPE was generally consistent and irrespective of races. Previous studies have consistently showed that MetS has a strong prompting effect on the prostate volume and/or prostate growth rates^32^,^33^. Interestingly, although the associations between MetS and LUTS in the general population was rather inconsistent, there was a significant amount of evidence indicated that MetS could still be positively associated with the severity of LUTS when there was a concomitant BPH/BPE in the same population^29^,^34^,^35^, and such associations were also valid in the general Asian population^36^,^37^. A possible explanation for this might be the effects of MetS on BPH are time-dependent or there existed a latency or induction period, thus the studies that performed on patients who had already developed BPH/BPE there a direct association could be more likely to detected, especially at the cross-sectional level.

To the best of our knowledge, this is the first prospective study showing evidence of MetS and its components as risk factors for BPH in a Chinese aging male population. Certain chronic diseases such as diabetes, CVD, subclinical ischemic stroke or autoimmune disease were tend to increase the clinic visit times and this could be a methodological explanation for the associations between MetS and clinical BPH development as we observed; however, considering our data on outcomes were largely collected by the routine health check-ups and all the entered subjects in our cohort should have an equal access to primary evaluation and therefore have an equal opportunity to be identified with clinical BPH, thus surveillance bias alone is unlikely to fully explain our results. Moreover, since the times of most failure events in our study were calculated at the end of the corresponding follow-up, the overall hazards of events were likely underestimated. Taken together, our prospective cohort confirmed the previous cross-sectional findings and further supports the notion that MetS could be an effective target for BPH prevention and intervention.

In the multivariate analyses, we found that central obesity and low HDL-C were the main drivers of the associations between MetS and the risk of clinical BPH, and the “significant” hazard ratios of central obesity were much more greater than that for low HDL-C. Our results are generally in line with a recently reported meta-analysis in which that the differences in PV were equally weighted as a factor of aging, central obesity and low HDL-C^32^. Although the exact nature and origins of the associations between MetS and BPH are still poorly understood, our specific findings highlighted the role of chronic inflammation in these associations. Not only was prostatic inflammation found to be associated with surgical BPH specimens^38^, but the degree and severity of inflammation were correlated with prostate enlargement^39^. In this context, our previous study in the same health check-ups program indicated that the elevated mean platelet volume, a surrogate marker of systemic inflammation, could also be a novel predictor of MetS-induced inflammation in the progression of BPH^40^.

In a former reported larger-scale multiethnic cohort, subjects with a BMI of <25.0, 25.0–29.9, and 30.0–34.9 kg/m^2^ had a median PV of 44.0 mL, 48.0 mL, and 52.0 mL. Furthermore, in the multivariable analyses, higher BMI was associated with larger PV (beta estimate 0.011; 95% CI 0.005–0.017; *p* < 0.001). Although the absolute differences in median PV between different BMI categories did not reach significance among their Southeast-Asian subgroup, the authors declared this may be because of the limited statistical power^41^; on the contrary, in a Chinese cohort reported by Xie *et al*.^42^, each 0.37 kg/m^2^ increment in BMI was associated with a 1 cm^3^ increase in PV. Adipose tissue, once considered a storage reservoir, is now regarded as a complex endocrine organ that responds to various afferent signals by secreting bioactive substances, which included resistin, leptin, tumor necrosis factor-α (TNF-α), interleukin (IL)-6, C-reactive protein (CRP), fibrinogen and plasminogen activator inhibitor (PAI-1)^43^. On the whole, these adipocytokines could induce insulin resistance and have proinflammatory effects. On the other hand, adiponectin (APN)-a circulating tissue-specific hormone, which stimulates glucose metabolism and fatty acid oxidation in the muscle, enhance insulin sensitivity in the liver, and inhibits macrophage transformation to foam cells within the vascular wall, is usually lowered in individuals with central obesity^44^.

At the biochemical level, obesity and particularly central obesity-induced macrophage infiltration in the adipose tissue is responsible for the secretion of the abovementioned adipocytokines and further leads to the activation of prostate-associated lymphoid tissue (PALT), which would then involved in the prostate enlargement^45^. Alternatively, as a protective effect of dihydrotestosterone (DHT) on inflammatory mediators has been observed in prostatic cells *in vitro*^46^, obesity-induced increased estrogen to testosterone ratio by enhanced activity of aromatase could also provide a reasonable explanation for the role of obesity in the course of BPH^47^. On the other hand, it has also been reported that certain MetS components, including dyslipidemia and especially the low HDL-C in circulation, could synergistically boost inflammation, tissue remodeling and consequent enlargement of the prostate^10^. Taken together, the findings of our study adds to the evidence that chronic inflammation is the main mechanism at the crossroad between MetS and BPH.

Our definitions of the main outcome, i.e. clinical BPH, deserve special discussion. Although there have been much epidemiological studies focusing on the effects of extrinsic risk factors on the risk of BPH, the lack of consensus about the definition of clinical BPH hampers the determination of putative risk factors of BPH^48^. Previously, the Krimpen Study Group had suggested that an I-PSS >7 could be a simple case definition of clinical BPH^49^; however, it should be noted that I-PSS does not essentially reflect the existing of BPH because it provides no information about the cause of urinary symptoms. By the contrast, in the present study we defined clinical BPH on the basis of a combination of a clinical diagnosis of BPH and having histories of specific treatment for BPH. Considering not all men with BPH develop LUTS, the lack of histological confirmation of BPH might also lead to underreporting of existed BPH and consequently the misclassification. However, this methodological effect of a less precise definition was unlikely to be differential with regards to the investigated risk factors and, thus, was expected to bias results toward the null, rather than caused spurious associations. In this context, it is possible that the lack of associations between the other MetS components examined and the risk of clinical BPH were also a result of such bias due to potential misclassification. On the other hand, analyses ended up in the same direction and of even stronger magnitudes aimed at the clinically significant BPH, further suggested that our definition of clinical BPH did not drive the results and the actual associations between MetS and BPH development might be even stronger than we report.

Alternatively, it is also well-known that the serum PSA levels serves as a reliable surrogate for PV and is therefore also helpful in deciding to which treatment a given patient may respond, thus the PSA assessment are regarded as “Recommended” in the European guideline^50^. Furthermore, a PSA of >1.4 ng/mL could be a proxy for a significant increase in prostate volume and a greater probability of benefit from 5-α reductase inhibitor (5-ARIs)^51^. However, our previous research in the same study population has clearly demonstrated that the presence of MetS was independently associated with an 11.3% decline in serum PSA levels compared with subjects without MetS in the general population, and the increasing number of MetS components was significantly and linearly associated with declining in serum PSA levels^52^; thus, using this cut-off value of PSA as an simple indicator of clinical BPH may underestimated the actual incidence, especially in subjects with MetS. Furthermore, since our institutional routine health check-ups were performed annually and former researches have reported that after 6 months of 5-ARIs treatment the serum PSA levels will be reduced by half 53, using a PSA of >1.4 ng/mL rather than the explicit history of medication treatment would also lower the detection rates of clinical BPH both in subjects with and without MetS. For these two main reasons, we defined clinical BPH as to *either* a clinical diagnosis of BPH *or* histories of specific treatment for BPH, which was closely similar to that used in recent epidemiological studies^23^,^24^,^25^.

Our study has numerous strengths, including the prospective design and data collection, the use of a large socioeconomically diverse cohort, the loss to follow-up was minimized, and our ability to control for a broad range of covariates. In particular, more than one-third (37.9%) of the subjects had experienced the outcome of interest (i.e. developed clinical BPH during the follow-up), which provided us with sufficient statistical power to identify any causal association between MetS and the risk of clinical BPH, as the prevalence of MetS at baseline in this study population was 22.9% (274/1,198). However, there were some limitations need to be noted when interpreting our results. Firstly, our study is limited geographically to the northern urban area in China, which may not be fully representative of the entire Chinese population, as well as the largely Han, well-educated and generally healthy nature of our study sample may restrict generalizability of our findings; however, the baseline prevalence of MetS in our cohort is comparable with the general Chinese population^54^, suggesting that there were likely no meaningful differential referral patterns based on the status of MetS. Given that our findings are generally in keeping with other population-based studies, the inferences that based on comparisons between MetS and BPH outcomes are likely valid, although future studies to address these associations directly in other populations, particularly in individuals of other races, are warranted. Secondly, there was a relatively long interval between measurements of exposures and ascertainments of outcomes and we do not know to what extent exposures may have changed subsequently. If the subjects changed exposure categories, this misclassification would weaken the associations between exposures and the clinical BPH development, although there is evidence that blood chemistries remain relatively stable over time^55^. Thirdly, PV was measured by using a suprapubic ultrasonography rather than the transrectal ultrasound (TRUS) in our health check-ups. Although the suprapubic ultrasonography was prone to slightly overestimate the prostate volume compared to TRUS^56^, it has been reported that there was a strong correlation between measurements of prostate volume measured by suprapubic ultrasonography and the real prostate volume in excised specimens^57^,^58^. Thus, the suprapubic ultrasonography was considered a more rapid, convenient and free from suffering examination in the setting of health check-ups, compared with the TRUS^59^. Furthermore, the methodological effect of a less precise estimate of PV was also unlikely to be differential with regards to the risk factors and was expected to bias the results toward null, rather than caused spurious associations between MetS and the risk of clinical BPH. Fourthly, we did not directly assessed whether treating MetS may prevent BPH; however, as we observed a stronger associations between MetS and the risk of the clinically significant BPH, an interesting finding which corresponding to our study aim, we may further speculated that the mechanisms may still exist after BPH development and could continuously affect the clinical progression of BPH. Finally, because we did not have the serial measurements on metabolic risk factors prior to study entry, it is currently inaccessible for us to evaluate the effect of duration of MetS has on the risk of BPH; however, considering the presence of MetS and the duration of MetS biologically associated and reflect similar risk factor profiles, our estimates of relative risk, which are the key findings of our study, should not be biased by this limitation, although the effect of duration of MetS has on the risk of BPH merits consideration for future studies.

## Conclusions

In this prospective cohort study of Chinese aging men, MetS is causally associated with increased risk of BPH. These findings suggest that in addition to a myriad of well established health benefits, maintaining a metabolic healthy body may also prevent clinical BPH development in aging men. On the whole, this study improves the understanding of the underlying biology of a potentially modifiable BPH risk factor and contributes to the knowledge base on MetS for future BPH prevention studies.

## Additional Information

**How to cite this article**: Zhao, S.-C. *et al*. Associations between metabolic syndrome and clinical benign prostatic hyperplasia in a northern urban Han Chinese population: A prospective cohort study. *Sci. Rep.* **6**, 33933; doi: 10.1038/srep33933 (2016).

## Figures and Tables

**Table 1:** Baseline characteristics grouped by metabolic syndrome status.

Characteristics^1^	Metabolic syndrome^1^				*p*-value
	Present (*n* = 258)		Absent (*n* = 872)		
*Socio-demographics*					
Age (year)	54.0 (51.0, 58.0)		52.0 (49.0, 57.0)		**0.038**^3^
Marital status					
married and living with spouse	255	98.8%	807	92.5%	**0.008**^4^
not married/widowed	3	1.2%	65	7.5%	
Socio-economic status					
lower 25% of the distribution	14	5.4%	180	20.6%	<**0.001**^4^
middle 50% of the distribution	153	59.3%	457	52.4%	
upper 25% of the distribution	91	35.3%	235	27.0%	
*Lifestyle factors*					
Smoking status					
never	5	1.9%	15	1.7%	0.445^4^
ex-user	6	2.3%	47	5.4%	
current-user	247	95.8%	810	92.9%	
Alcohol consumption					
never	16	6.2%	51	5.8%	0.873^4^
ex-user	163	63.2%	548	62.8%	
current-user	79	30.6%	273	31.4%	
Physical activity					
low (less than 100)	216	83.7%	664	76.1%	0.126^4^
medium (100–249)	37	14.3%	192	22.0%	
high (250 or greater)	5	1.9%	16	1.9%	
Sexual activity					
inactive	109	42.2%	268	30.7%	**0.029**^4^
active	149	57.8%	604	69.3%	
*BPH components measurements*					
PV (cm^3^)	18.1 (15.3, 22.5)		18.1 (15.4, 20.8)		0.868^3^
Q_max_ (mL/s)	18.8 (14.9, 22.5)		18.9 (16.8, 23.0)		0.573^3^
Serum PSA (ng/mL)	1.42 ± 1.24		1.49 ± 1.12		0.479^5^
IPSS score (besides the QoL)	2.0 (1.0, 4.0)		2.0 (1.0, 4.0)		0.938^3^
QoL score	1.0 (1.0, 2.0)		1.0 (1.0, 2.0)		0.966^3^
*Metabolic components*					
Central obesity (cm)^2^	92.5 (90.0, 94.0)		89.0 (84.0, 94.0)		<**0.001**^3^
positive	204	79.1%	423	48.5%	<**0.001**^4^
negative	54	20.9%	449	51.5%	
Elevated SBP (mm Hg)^2^	126.0 (119.5, 140.0)		128.0 (118.0, 136.0)		0.778^3^
positive	100	38.8%	367	42.1%	0.425^4^
negative	158	61.2%	505	57.9%	
Elevated DBP (mm Hg)^2^	82.0 (74.0, 84.0)		81.0 (76.0, 87.0)		0.511^3^
positive	54	20.9%	307	35.2%	**0.009**^4^
negative	204	79.1%	565	64.8%	
Hypertension^2^					
positive	97	37.6%	440	50.5%	**0.016**^4^
negative	161	62.4%	432	49.5%	
IFG (mg/dL)^2^	89.7 (85.8, 104.5)		90.5 (85.1, 96.4)		0.934^3^
positive	68	26.4%	103	11.8%	**0.010**^4^
negative	190	73.6%	769	88.2%	
Hypertriglyceridemia (mg/dL)^2^	137.8 (87.8, 182.0)		114.7 (81.2, 161.5)		**0.014**^3^
positive	121	46.9%	252	28.9%	<**0.001**^4^
negative	137	53.1%	620	71.1%	
Low HDL-C (mg/dL)^2^	46.4 (37.2, 52.4)		49.0 (43.2, 56.2)		<**0.001**^3^
positive	100	38.8%	79	9.1%	<**0.001**^4^
negative	158	61.2%	793	90.9%	
LDL-C (mg/dL)	111.2 ± 34.1		112.5 ± 28.5		0.559^5^
Total cholesterol (mg/dL)	180.1 ± 34.3		184.5 ± 32.7		0.147^5^
BMI (kg/m^2^)	26.7 ± 2.6		25.4 ± 2.6		<**0.001**^5^
*Comorbidities*					
Diabetes					
positive	71	27.5%	120	13.8%	<**0.001**^4^
negative	187	72.5%	752	86.2%	
CVD					
positive	50	19.4%	76	8.7%	<**0.001**^4^
negative	208	80.6%	796	91.3%	
Subclinical ischemic stroke					
positive	31	12.0%	94	10.8%	0.578^4^
negative	227	88.0%	778	89.2%	
Autoimmune disease					
positive	17	6.6%	49	5.6%	0.560^4^
negative	241	93.4	823	94.4%	

Abbreviations: BPH = benign prostatic hyperplasia; SBP = systolic blood pressure; DBP = diastolic blood pressure; IFG = impaired fasting glucose; HDL-C = high-density lipoprotein cholesterol; LDL-C = low-density lipoprotein cholesterol; PV = prostate volume; Q_max_ = maximum urinary flow rate; IPSS = International Prostate Symptom Score; QoL = Quality of life; PSA = prostate-specific antigen; BMI = body mass index; CVD = congestive heart disease.

^1^Data are number and percentage, median (25th and 75th quartile), or mean ± S.D.

^2^Criteria for the positive individual components of the metabolic syndrome were defined by the statement from the International Diabetes Federation Task Force on Epidemiology and Prevention; National Heart, Lung, and Blood Institute; American Heart Association; World Heart Federation; International Atherosclerosis Society; and International Association for the Study of Obesity (Alberti *et al*.)^6^.

^3^Mann-Whitney U test.

^4^Pearson chi-square test.

^5^student-*t* test. Bold indicates statistically significant.

**Table 2:** The prevalence of metabolic syndrome and its components at baseline and the incidence of clinical benign prostatic hyperplasia at follow-up according to baseline age categories.

Outcomes	baseline age categories					*p*-value^5^
	Total	45–49	50–54	55–59	≥60	
Total	1,130	316	371	300	143	—
MetS^1^	258	47 (14.9%)	83 (22.4%)	107 (35.7%)	21 (14.7%)	<0.001
Central obesity^1^	627	160 (50.6%)	224 (60.4%)	184 (61.3%)	59 (41.3%)	<0.001
Hypertension^1^	537	113 (35.8%)	153 (41.2%)	174 (58%)	97 (67.8%)	<0.001
IFG^1^	171	43 (13.6%)	26 (7.0%)	81 (27%)	21 (14.7%)	<0.001
Hypertriglyceridemia^1^	373	72 (22.8%)	132 (35.6%)	126 (42%)	43 (30.1%)	<0.001
Low HDL-C^1^	179	40 (12.7%)	49 (13.2%)	75 (25.0%)	13 (9.1%)	<0.001
Clinical diagnosis of BPH^2^	259	58 (18.4%)	85 (22.9%)	73 (24.3%)	43 (30.1%)	<0.001
Received medication for BPH^3^	109	14 (4.4%)	26 (7.0%)	34 (11.3%)	35 (24.5%)	<0.001
Undergone surgery for BPH^3^	61	4 (1.3%)	8 (2.2%)	20 (6.7%)	29 (20.3%)	<0.001
Overall clinical BPH^4^	429	76 (24.1%)	119 (32.1%)	127 (42.3%)	107 (74.8%)	<0.001

Abbreviations: MetS = metabolic syndrome; IFG = impaired fasting glucose; HDL-C = high-density lipoprotein cholesterol; BPH = benign prostatic hyperplasia.

^1^Criteria for metabolic syndrome and the positive individual components of the metabolic syndrome were defined by the statement from the International Diabetes Federation Task Force on Epidemiology and Prevention; National Heart, Lung, and Blood Institute; American Heart Association; World Heart Federation; International Atherosclerosis Society; and International Association for the Study of Obesity (Alberti *et al*.)^6^.

^2^Criteria for the clinical diagnosis of BPH was defined by the statement from the 2011 Chinese Guideline for BPH.

^3^The defined cases of “clinically significant BPH”.

^4^The overall clinical BPH was defined as to *either* a clinical diagnosis of BPH *or* histories of specific treatment for BPH.

^5^Mantel-Haenszel extension test for trend. Bold indicates statistically significant.

**Table 3:** Age-adjusted and multivariable associations of metabolic syndrome with the risk of clinical benign prostatic hyperplasia.

	Clinical BPH Row % (*n*/total)	Non-BPH Row % (*n*/total)	*p*-value^3^	HR (95% CI)	*p*-value	Multivariate-adjusted HR (95% CI)	*p*-value^4^
***Overall clinical BPH***^1^							
Dichotomously defined							
Non-MetS	32.9 (287/872)	67.1 (585/872)	<**0.001**	Ref	Ref	Ref	Ref
MetS	55.0 (142/258)	45.0 (116/258)		1.38 (1.18–1.58)	**0.001**	1.29 (1.08–1.50)	<**0.001**
No. of metabolic risk factors							
0 components	19.9 (38/191)	80.1 (153/191)	<**0.001**	Ref	Ref	Ref	Ref
1 components	27.5 (93/338)	72.5 (245/338)		0.90 (0.71–1.09)	0.98	0.88 (0.67–1.09)	0.86
2 components	39.7 (136/343)	60.3 (207/343)		1.19 (0.93–1.45)	0.55	1.18 (0.89–1.47)	0.29
≥3 components (i.e. MetS)	62.8 (162/258)	37.2 (96/258)		1.42 (1.10–1.74)	**0.035**	1.37 (1.08–1.66)	**0.014**
***Clinically significant BPH***^2^							
Dichotomously defined							
Non-MetS	12.5 (109/872)	87.5 (763/872)	<**0.001**	Ref	Ref	Ref	Ref
MetS	23.6 (61/258)	76.3 (197/258)		1.54 (1.33–1.75)	<**0.001**	1.49 (1.27–1.71)	<**0.001**
No. of metabolic risk factors							
0 components	10.5 (20/191)	89.5 (171/191)	**0.001**	Ref	Ref	Ref	Ref
1 components	11.8 (40/338)	88.2 (298/338)		1.08 (0.85–1.31)	0.85	1.02 (0.85–1.19)	0.84
2 components	14.9 (51/343)	85.1 (292/343)		1.38 (1.12–1.64)	0.069	1.31 (1.08–1.54)	**0.039**
≥3 components (i.e. MetS)	22.9 (59/258)	77.1 (199/258)		1.64 (1.33–1.95)	**0.015**	1.58 (1.26–1.90)	**0.001**

Abbreviations: HR = hazard ratio; CI = confidence interval; BPH = benign prostatic hyperplasia; MetS = metabolic syndrome.

^1^The overall clinical BPH is defined as to *either* a clinical diagnosis of BPH *or* histories of specific treatment for BPH.

^2^The clinically significant BPH is defined as having histories of specific treatment for BPH.

^3^Pearson chi-square test or Mantel-Haenszel extension test for trend as appropriate.

^4^Multivariable Cox proportional hazards models to adjust for the following confounding factors: age, marital status, socio-economic status, smoking history, alcohol usage, physical activity, sexual activity, BMI, total cholesterol, LDL-C, comorbidities which included diabetes, CVD, subclinical ischemic stroke and autoimmune disease, and the baseline BPH components measurements which included PV, Q_max_, IPSS and serum PSA levels. Bold indicates statistically significant.

**Table 4 t4:** Age-adjusted and multivariable associations of the individual components of metabolic syndrome with the risk of clinical benign prostatic hyperplasia.

Individual metabolic risk factors	Age-adjusted HR (95% CI)	*p*-value^4^	Multivariate-adjusted HR (95% CI)	*p*-value^4^
***The overall clinical BPH***^1^				
Central obesity^3^	2.01 (1.23–2.79)	**0.026**	1.93 (1.14–2.72)	**0.001**
Hypertension^3^	0.79 (0.56–1.02)	0.387	0.75 (0.54–0.96)	0.223
IFG^3^	1.07 (0.76–1.38)	0.523	1.05 (0.68–1.42)	0.493
Hypertriglyceridemia^3^	1.09 (0.80–1.38)	0.735	1.04 (0.72–1.36)	0.687
Low HDL-C^3^	1.63 (1.14–2.12)	**0.039**	1.56 (1.08–2.04)	**0.012**
***Clinically significant BPH***^2^				
Central obesity^3^	2.29 (1.48–3.10)	**0.011**	1.98 (1.20–2.76)	<**0.001**
Hypertension^3^	0.87 (0.68–1.06)	0.275	0.81 (0.63–0.99)	0.182
IFG^3^	1.15 (0.81–1.49)	0.469	1.10 (0.83–1.37)	0.402
Hypertriglyceridemia^3^	1.14 (0.82–1.46)	0.664	1.08 (0.74–1.42)	0.546
Low HDL-C^3^	1.71 (1.20–2.22)	**0.029**	1.64 (1.05–2.23)	**0.003**

^1^The overall clinical BPH was defined as to *either* a clinical diagnosis of BPH *or* histories of specific treatment for BPH.

^2^The clinically significant BPH was defined as having histories of specific treatment for BPH.

^3^Criteria for the positive individual components of the metabolic syndrome were defined by the statement from the International Diabetes Federation Task Force on Epidemiology and Prevention; National Heart, Lung, and Blood Institute; American Heart Association; World Heart Federation; International Atherosclerosis Society; and International Association for the Study of Obesity (Alberti *et al*.)^6^.

^4^Multivariable Cox proportional hazards models included all the dichotomous components of metabolic syndrome in a mutually adjusted state for each and also adjusted for the other confounding factors: age, marital status, socio-economic status, smoking history, alcohol usage, physical activity, sexual activity, BMI, total cholesterol, LDL-C, comorbidities which included diabetes, CVD, subclinical ischemic stroke and autoimmune disease, and the baseline BPH components measurements which included PV, Q_max_, IPSS and serum PSA levels. Bold indicates statistically significant.
